# Radiation-induced gastric injury during radiotherapy: molecular mechanisms and clinical treatment

**DOI:** 10.1093/jrr/rrad071

**Published:** 2023-10-03

**Authors:** Guangxia Chen, Zuxiang Yu, Yuehua Zhang, Shiyu Liu, Chong Chen, Shuyu Zhang

**Affiliations:** Department of Gastroenterology, The First People’s Hospital of Xuzhou, Xuzhou Municipal Hospital Affiliated to Xuzhou Medical University, Xuzhou 221200, China; Laboratory of Radiation Medicine, West China School of Basic Medical Sciences and Forensic Medicine, Sichuan University, Chengdu 610041, China; Laboratory of Radiation Medicine, West China School of Basic Medical Sciences and Forensic Medicine, Sichuan University, Chengdu 610041, China; Department of Gastroenterology, The First People’s Hospital of Xuzhou, Xuzhou Municipal Hospital Affiliated to Xuzhou Medical University, Xuzhou 221200, China; Department of Gastroenterology, The First People’s Hospital of Xuzhou, Xuzhou Municipal Hospital Affiliated to Xuzhou Medical University, Xuzhou 221200, China; Laboratory of Radiation Medicine, West China School of Basic Medical Sciences and Forensic Medicine, Sichuan University, Chengdu 610041, China; Department of Nuclear Medicine, The Second Affiliated Hospital of Chengdu Medical College, China National Nuclear Corporation 416 Hospital , Chengdu 610051, China; NHC Key Laboratory of Nuclear Technology Medical Transformation (Mianyang Central Hospital), Mianyang 621099, China

**Keywords:** ionizing radiation, radiotherapy (RT), radiation-induced gastric injury (RIGI), diagnosis, treatment

## Abstract

Radiotherapy (RT) has been the standard of care for treating a multitude of cancer types. Radiation-induced gastric injury (RIGI) is a common complication of RT for thoracic and abdominal tumors. It manifests acutely as radiation gastritis or gastric ulcers, and chronically as chronic atrophic gastritis or intestinal metaplasia. In recent years, studies have shown that intracellular signals such as oxidative stress response, p38/MAPK pathway and transforming growth factor-β signaling pathway are involved in the progression of RIGI. This review also summarized the risk factors, diagnosis and treatment of this disease. However, the root of therapeutic challenges lies in the incomplete understanding of the mechanisms. Here, we also highlight the potential mechanistic, diagnostic and therapeutic directions of RIGI.

## INTRODUCTION

Cancer is a leading cause of death and a major obstacle to increasing life expectancy in countries worldwide. In 2020, there were expected to be 19.3 million new cases of cancer, and about 10 million cancer patients died [1]. Radiotherapy (RT), surgery and chemotherapy are the three main approaches to the treatment of malignant tumors. Approximately 60% of cancer patients receive RT at different stages of their treatment, either alone or together with other treatments [2]. However, RT will inevitably cause damage to adjacent normal tissues or organs, which is the main limitation of radiation therapy efficacy.

Radiation-induced gastric injury (RIGI) refers to gastric tissue damage caused by the cumulative dose of radiation exceeding the biological effect threshold. It is a common complication of RT for thoracic and abdominal tumors, such as gastric, esophageal and hepatocellular cancers. In the acute phase, patients often present with abdominal pain, abdominal distension and possibly even bloody stools. However, high doses of radiation, as might be received in a nuclear accident, can almost deplete gastric mucosal epithelial cells [3]. In the chronic phase, indigestion and long-term abdominal vague pain are the main symptoms. With increased irradiation, the normal gastric mucosa gradually shows chronic atrophic changes [4–7]. Patients with a history of radiation therapy for thoracic and abdominal tumors can be clinically diagnosed with RIGI by gastroscopy, barium meal of the gastrointestinal tract and abdominal computed X-ray tomography (CT). Serum gastritis markers (Gastrin 17, Pepsinogen I and II) can be invoked as auxiliary diagnostic and predictive indicators. Patients with mild cases are usually given antacids and gastric mucosal protectors, while patients with gastric ulcers and bleeding require emergency gastroscopy hemostasis or hospitalization for comprehensive treatment. The estimated incidence of RIGI varies widely across studies. Physicians’ unfamiliarity with radiation types and RT risk factors in clinical practice can likewise increase a patient’s risk of RIGI.

## PATHOLOGICAL BASIS AND MOLECULAR MECHANISM OF RIGI

Gastric is the primary organ in the digestive system. It stores food bolus from the esophagus through receptive relaxation, initially digests food through the secretion of gastric acid and pepsin, and facilitates peristalsis of the gastric parietal until gastric emptying. Additionally, the gastric secretes intrinsic factor to promote the absorption of vitamin B12. The core damage mode of ionizing radiation is the ionization of intracellular molecules or the ejection of electrons. Mechanisms of RIGI include direct injury, indirect injury and bystander effect.

### Direct injury

Direct injury means that ionizing radiation breaks chemical bonds and knocks out electrons. Most molecules (such as mRNA and proteins) can be rapidly renewed, so the exact damage effect is relatively weak. DNA is the largest molecule in cells, and it has a strict replication process and a slow renewal rate. It is the target area directly damaged by radiation and the main cause of cell death. The main types of radiation-induced DNA damage include bases and ribose damage, single-strand breaks, double-strand breaks (DSBs) and clustered DNA damage [8]. DSBs are the main damage factor of radiation cytotoxicity. DNA damage response determines the outcome of cell damage through a set of closely related signaling pathways, which are divided into two parts: receptors and effectors. H2AX phosphorylation occurs 5–30 min after DSBs. Then, dozens of proteins are recruited to the DSBs region to form a ‘focal point (IRIF)’ that transmits signals to effectors. There are at least three pathways of effector: cell death, cell cycle arrest and repair of broken DNA. The regeneration of gastric mucosal epithelial cells is fast; however, during the repair process of DSBs, it can easily lead to dangerous mutations and deletions of genes, increasing radiosensitivity. Therefore, the cell death mechanism appears to be more favorable for protecting gastric mucosa than repairing DNA damage. Excessive cell death of cells may require rapid proliferation and differentiation of stem cells to replenish them, otherwise the structure and normal function of the organ will be affected [9]. Similar to radiation intestinal injury, studies have reported that gastric antrum stem cells produce a large number of normal epithelial cells after RIGI to maintain the normal function of gastric mucosal epithelium [10].

### Indirect injury

**Fig. 1:**
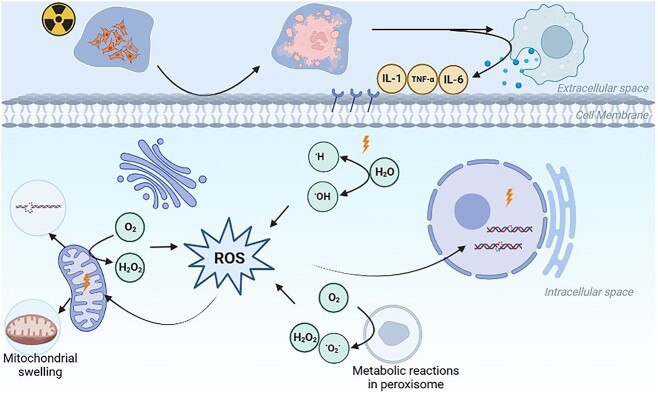
Potential mechanisms of RIGI. Ionizing radiation directly breaks the DNA and protein, leading to the production of a large number of ROS in mitochondria and cytoplasm after exposure to radiation. Excess ROS can also damage DNA and protein. These changes cause the mutation, aging and death. Mononuclear macrophages clean the aging and dead cells and release inflammatory cytokines (IL-1, IL-6, TNF-α), and these cytokines accelerate the death of irradiated cells.

Water is the main constituent of cells, and the average water content of the cells was about 60–90%. The indirect effects of ionizing radiation refer to radiation causing the excitation and ionization of water, resulting in the generation of a burst of free radicals and reactive oxygen species (ROS). These highly unstable small molecules subsequently attack macromolecular substances such as nucleic acids, proteins and lipids (Fig. 1). Physiological levels ROS are by-products of natural cellular metabolism, which have a benefit to a variety of physiological processes, including killing invading pathogens, promoting wound healing and repairing tissues [11]. Normal levels of ROS are metabolized by a variety of enzymes and antioxidant small molecules in cells [12]. Short-term and long-term oxidative stress reactions dominated by mitochondrial dysfunction after ionizing radiation generate enormous amounts of ROS, which disturb the balance of oxidative and antioxidant systems in cells, especially in the gastrointestinal tract. Since ROS are highly reactive with intracellular proteins, lipids, carbohydrates and nucleic acids, excessive ROS production can not only lead to apoptosis and necrosis caused by DNA damage [13], but can also lead to mitochondrial membrane damage and increased permeability, prompting the release of cytochrome C (a mitochondria-dependent pro-apoptotic molecule) into the cytoplasm, activating the Caspase-9-dependent apoptosis pathway and causing apoptosis in affected cells, resulting in increased apoptosis of gastric mucosal epithelial cells and vascular endothelial cells, and a decreased cell division rate. Finally，adverse clinical events such as gastric mucosal erosion, ulceration and eventually gastric mucosal atrophy and intestinal epithelial metaplasia are induced. In dying cells, interleukin-1 (IL-1), IL-6, IL-8, IL-13, IL-33, tumor necrosis factor-α (TNF-α) and other inflammatory cytokines are released by pathways such as NF-κB and STAT. These cytokines activate the redox system and further increase ROS production [14, 15]. The damage caused by ROS is not only evident in irradiated cells, but also in the progeny of these cells [16].

**Table 1:** Risk factors for RIGI

**Radiation risk factors**
Total gastric dose, >45 Gy % Total gastric volume receiving ≥50 Gy (V50), > 16% % Total gastric volume receiving ≥25 Gy (V25), >6.3% Irradiation of gastric antrum Combined chemotherapy **Host risk factors**
Main portal vein tumor thrombosis Cirrhosis and portal hypertension
History of upper gastrointestinal bleeding

### Bystander effect

Radiation-induced intracellular oxidative damage not only occurs in irradiated cells, but may also spread the effects to adjacent nonirradiated cells through signal transduction. These adjacent cells and filial cells manifest the same biological changes as the irradiated cells, a phenomenon defined as the bystander effect of radiation [17, 18]. Specifically, when 1% of cells are irradiated, about 30% of cells will manifest the same toxic radiation effects [19]. These effects include chromosomal aberrations, sister chromatid exchanges, genetic instability, altered gene expression, apoptosis, signal transduction alterations, radiation adaptive response, ROS production and tumor transformation [20, 21]. These effects can be exerted within a range of 1–5 mm from the irradiation distance [20, 22]. Irradiated cells secrete a large number of bystander signaling mediators, and these signaling mediators stimulate the production of ROS/RNS, cytokines and chemokines, oxidases and other inflammatory factors [23]. These signaling mediators may propagate to bystander cells through direct cell-to-cell contacts, gap junctions [24] and exosomes [25].

### Candidate molecules of RIGI

The persistent inflammatory response in the stomach is the principal manifestation of radiation damage, which leads to structural damage and dysfunction, even organ failure and primary cancer formation. However, the molecules driving these inflammations remain unclear. Activation of the p38/MAPK pathway significantly increased gastric mucosal epithelial cell injury, whereas microtubule-associated serine/threonine kinase 1 protected against cellular radiation injury by inhibiting activation of this pathway and reducing the levels of BAX, cleaved Caspase3, Cleaved PARP1 and increasing the expression of BCL2 [26]. In addition to apoptosis, pyroptosis is another mechanism involved in radiation-mediated gastric injury. Knockdown of GSDME, a key factor in pyroptosis, was shown to reduce RIGI [27]. In a previous study [28], mouse stomach was irradiated with 6 or 12 Gy X-ray irradiation and we checked the mRNA and lncRNA expression. The results revealed that 17 mRNAs were upregulated, and 10 mRNAs were downregulated consistently in gastric tissues irradiated with 6 and 12 Gy, including D site-binding protein and fibrinogen-like protein 1 (Fgl1). Thirteen upregulated and 96 downregulated lncRNAs were commonly changed in 6 and 12 Gy irradiated gastric tissues, and these common changes may illustrate the molecular changes during RIGI. In another study, a custom oligonucleotide array of 208 genes was used to compare the gene expression profiles of gastric cancer between atomic-bomb survivors and nonexposed patients. The expression of versican and osteonectin was significantly lower in the Stage I/II gastric cancer tissues of atomic-bomb survivors. Because both versican and osteonectin can be induced by transforming growth factor (TGF)-β1, they attributed this alteration to changes in TGF-β signaling pathway [29].

## RISK FACTORS OF RIGI

According to the functional subunits, gastric is a ‘parallel organ’. Usually, the single-point high-dose irradiation does not have enough power to destroy the structure and function of the gastric as thoroughly as the ‘serial organs’ (such as the spinal cord), while the dose-volume effect is particularly prominent in RIGI. Therefore, radiation oncologists attempt to limit RIGI by minimizing the total dose for a certain irradiated volume. It was reported that when carbon ion RT for pancreatic cancer was performed with D2 cm^3^ < 46Gy (RBE), the incidence of gastric ulcers was reduced by half compared with other carbon ion RT [30, 31], and the total dose of the former (55.2 Gy (RBE) in 12 fractions) was greater than that of the latter (30–36.8 Gy equivalents (GyE) in 8 fractions). When the cumulative dose was determined, upper gastrointestinal toxicity was positively correlated with gastric exposure volume and defined the acute gastric critical volume for toxicity [32–34]. To determine the predictors of gastric injury after RT for hepatocellular carcinoma (HCC), a study observed pathological alterations of gastric after RT through gastroscopy, indicating that erosive gastritis and gastric ulcers are significantly related to dose-volume effects, when RT was delivered as 30–50 Gy (median 37.5 Gy) in 2–5 Gy (median 3.5 Gy) per fraction, V25 > 6.3% refers to a high incidence of gastric toxicity [35]. Fractional irradiation reduced the incidence and severity of acute gastric toxicity to a certain extent, but because the cumulative dose had no change, the advanced gastric toxicity was not attenuated [36, 37]. In addition, irradiation of the gastric antrum may increase the risk of gastric injury. Evidence from these observational clinical RT studies is necessary in order to refine the assessment of RIGI. Patients receiving RT usually undergo other combined therapies (like chemotherapy), and gastric mucosa protection drugs are often required during chemotherapy. It is obvious that combined chemotherapy could aggravate the burden of gastric [38]. Both cirrhotic portal hypertension and history of upper gastrointestinal bleeding increase the incidence of RIGI, especially hemorrhagic gastritis. The common drugs [non-steroidal anti-inflammatory drugs, glucocorticoid (GC)] that cause gastric ulcer and HP infection have little correlation with the occurrence of gastric ulcer and bleeding after RT [4, 39].

## DIAGNOSIS OF RIGI

### Clinical features

**Fig. 2:**
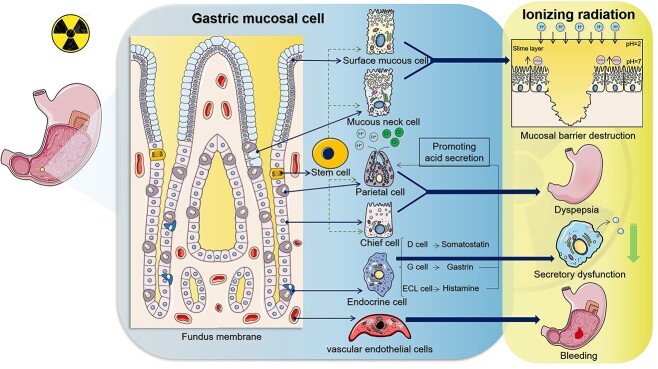
Radiogenic gastric injury and corresponding clinical manifestations. Gastric tissue consists of a variety of cells. The radiation breaks the mucosal barrier by damaging the surface mucous cells and mucous neck cells. The injury of parietal cells and chief cells leads to the dyspepsia. Vascular endothelial cell injury increases the incidence of gastric bleeding.

Till now, there are no clinical data of the incidence of RIGI when RT has been applied. To distinguish RIGI from other gastric pathology, checking the digestive symptoms and cancer or RT history is significant. The severity of RIGI varies from endoscopic findings with no clinical symptoms to life-threatening disease requiring hospitalization. Even if erosions and shallow ulcers have occurred, more than half of the patients may experience no symptoms or only mild intermittent abdominal pain. A physical examination of a patient with RIGI may yield normal results. Rarely, tarry stool occurs when the submucosal vessels are injured. Due to the small amount of bleeding, vomiting of blood usually does not occur, which is different from the upper gastrointestinal bleeding caused by esophageal and gastric varicose bleeding. Gastroscopy is an important basis for diagnosing RIGI. Changes such as mucosal erosion, patchy redness and ulcer formation can be observed. Severe ulcer can develop into gastric perforation, leading to symptoms of peritonitis will be founded [40] (Fig. 2).

### Endoscopy and imaging diagnosis

Although the symptoms of upper digestive tract may be vague, if a patient presents with these symptoms weeks or months following the completion of RT, physicians should also be suspicious of RIGI and promptly begin a diagnostic evaluation. Gastroscopy diagnosis is quite visual and reliable and is the cornerstone of clinical treatment and prognostic evaluation, but the sensitivity is low. According to the findings of gastroscopy and pathological biopsy at different time periods, the period within 3 months after RT is often defined as the acute phase and subacute phase, while periods exceeding 3 months are regarded as the chronic phase. Radiation gastritis is defined endoscopically as a new gastric mucosal lesion in the radiation-affected area [41]. Acute radiation gastritis most often occurs in the gastric antrum, usually within 3 weeks after the end of RT, nausea and vomiting are the predominant symptoms in this phase. The typical endoscopic manifestations include new or worsened dark red spots and patches around the gastric antrum, as well as exudative changes and ulcers [42]. Histologically, submucosal edema can be seen at the lesion site, with the gastric mucosa showing inflammation in the lamina propria, atrophy of epithelial cells, degeneration of parietal cells and chief cells and regenerated glands. However, imaging studies showed a normal gastric size [43]. Weight loss occurs in the subacute and chronic phases and can reach 25% of total body weight. Under the endoscope, the irradiated area shows the fusion of red plaques, flattening of folds, annular ulcers and telangiectasia, and persistent ulcers may be complicated by perforation and bleeding [41, 42]. Histologically, a large area of normal gastric epithelium has been replaced by stratified squamous epithelium. The lamina propria, muscularis mucosa and submucosa were destroyed, and regenerated glands consist exclusively of PAS-positive cells. CT showed thickening of the gastric wall and narrowing of the gastric cavity [43, 44]. Capillary dilatation is a gastroscopic typical manifestation of hemorrhagic gastritis [45], which is a serious complication.

## TREATMENT OF RIGI

### Drugs and vitamins

#### Proton pump inhibitors and other adjuvant drugs

Radiation protection and post-damage treatment have always been difficult in clinical radiobiology research. The development of new drugs, effective physical protection equipment, improvements in dose rates [46] and the search for the radiation protection effects using available clinical drugs [47] hold certain promise. However, there is still a long way to go before achieving complete clinical translation. At present, the clinical treatment of RIGI focuses on protecting the gastric mucosa and inhibiting the erosion of the radiation-injured mucosa by gastric acid. Proton pump inhibitors (PPIs) are the first-line treatment drugs for reflux disease, peptic ulcer and *Helicobacter pylori* (*H. pylori*) infection [48–50]. Omeprazole, pantoprazole, esomeprazole, lansoprazole and rabeprazole are some of the most commonly used. Several clinical studies have shown that the pharmacological activity of PPIs is not limited to the inhibition of gastric acid. The emerging effects of PPIs include reducing neutrophil aggregation and the permeability of mast cell [51], reducing inflammatory cytokine release [52] and regulating oxidative stress by scavenging ROS and free radicals [53–55]. Many studies have reported the positive effect of PPIs in oncology RT. PPIs increase radiosensitivity of breast cancer [56] and pancreatic cancer [57] by targeted inhibition of fatty acid synthase [58–60]. They also increase the radiosensitivity of head and neck squamous cell carcinomas by inhibiting cyclin-dependent kinases (Cdks) [61]. In addition, PPIs may also have a protective effect on normal tissues in the irradiated area by reducing the inflammatory response [62, 63]. These new effects seem to take PPIs to a new level and are easily linked to radiation protection. However, some large prospective cohort studies have revealed the long-term risks of PPIs. Long-term use of PPIs may impair the function of specific tissues, such as the cardiovascular system [64], kidney [65] and nervous system [66], and may increase the incidence of cancer [67]. However, the underlying mechanism is unclear and may be associated with impaired intracellular lysosomal activity [68]. Although the long-term adverse events of PPIs are of serious concern, the short-term use of PPIs has no obvious unsafe manifestations. Therefore, PPIs are still effective drugs for the treatment of radiation gastritis in clinical [69]. However, when using PPIs in combination with certain drugs, attention should be paid to drug interactions, especially the dissolution, absorption of pH-dependent antineoplastic [70]. For example, oral CDK4/6 inhibitor Palbociclib has been shown to improve the overall survival of breast cancer patients and enhance the radiosensitivity of HCC, cholangiocarcinoma [71–73] and lung cancer [74]. Studies have suggested that the combination of PPIs and Palbociclib reduces the clinical prognosis of breast cancer patients [70]. Whether PPIs attenuate the radiation sensitization effect of Palbociclib needs further investigation. When clinicians try to prescribe PPIs to these patients, such influences need to be taken into account. In addition to PPIs, it is important to be mindful of pH changes and influences of drug interactions when using commonly used acids, such as cimetidine, ranitidine and famotidine. Gastric mucosal protective agents and gastric motility drugs have proven effective in repairing gastric mucosal damage, restoring gastric motility and improving gastrointestinal symptoms (such as vomiting and abdominal pains). They also aid in restoring gastric digestive function. Furthermore, some dietary amino acids such as glutamine [75] and sperm amino acid [76] have a certain effect on the repair of gastric mucosal damage induced by radiation.

#### Antioxidants

Antioxidants confer radioprotection by reducing oxidative stress level in cells after irradiation. Although the radioprotective effects of various antioxidants on the gastrointestinal tract have been widely studied [77], few drugs or natural extracts have entered clinical trials or approved for radiation gastric injury prevention. Vitamin A, vitamin C and vitamin E are natural antioxidants, which exist in various foods and play a protective role in the survival of cells after radiation damage [78–81]. Oral preparations of these vitamins are also readily available.

#### GCs

GC is a class of steroid hormones secreted by the adrenal cortex fasciculus, which can regulate the biosynthesis and metabolism of carbohydrates, fats and proteins. It also shows an anti-inflammatory effect. GC has sporadic clinical trial evidence in the treatment of radiation enteritis [82, 83]. Similarly, there are only few case reports of successful use of prednisolone in the treatment of radiation hemorrhagic gastritis [45], which is attributed to its anti-inflammatory effect, including decreasing chemotaxis of monocytes and neutrophils, inhibiting adhesive molecule synthesis and decreasing eicosanoid production. As a result, gastric mucosa damage and degeneration are reduced. Besides, GC itself has the ability to enter the secretion of gastric acid and pepsin, while inhibiting mucus secretion, potentially inducing or aggravating radiation gastritis and gastric ulcers.

### Endoscopic and surgical treatment

Patients with mild gastric radiation injury are generally treated conservatively with drugs, but endoscopic treatment of severe ulcers and hemorrhagic gastritis is effective, especially for patients with liver cirrhosis. Argon plasma coagulation (APC) is a routine method for endoscopic treatment of hemorrhagic gastritis, which can stop bleeding repeatedly [41, 84]. Surgery is required for patients who need to take anticoagulants during the same period, and patients who have failed endoscopic treatments such as APC and spraying hemostatic powder [84].

### Stem cell-based therapy

**Fig. 3:**
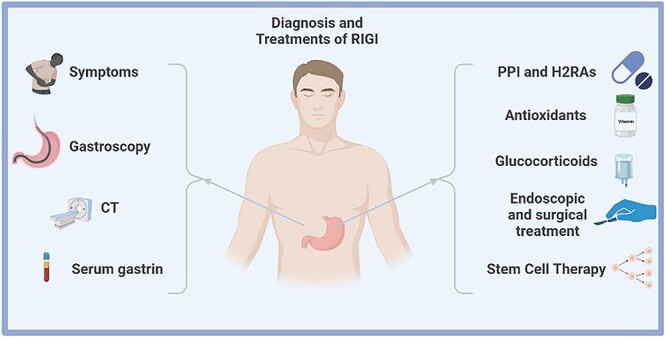
Diagnosis and treatments of RIGI. A RIGI patient usually has digestive symptoms. Gastroscope can directly observe the gastric lesions, while CT helps to distinguish other upper abdominal diseases. Serum markers of gastric inflammation (gastrin and pepsinogen) reflect the inflammation of gastric. PPIs and H2RAs are the first-line drugs for gastritis and ulcer. Antioxidants have a benefit on reducing oxidative stress. GC may have an anti-inflammatory effect on RIGI. Endoscopic treatment is appropriate in severe ulcers and hemorrhagic gastritis, while surgery is required for patients who need to take anticoagulants during the same period. Stem cell-based therapy is a new clue for the treatment of RIGI.

Mesenchymal stem cells (MSCs) are adult pluripotent stem cells that mainly exist in bone marrow, but also in skeletal muscle and other tissues. They not only have the potential to differentiate into a variety of cells, but also have the ability to secrete cytokines and migrate to damaged tissues. The isolation, purification and *in vitro* culture techniques of MSCs are relatively mature. It is reported that MSCs can improve the immune rejection of bone marrow transplantation [85], radiation-induced lung injury [86, 87] and ischemic cardiomyopathy [88]. In radiation-induced enteritis, MSCs protect epithelial stem cells [89]. One of the underlying mechanism of MSCs involves the activation of the Wnt/β-catenin signaling pathway via cytokines to increase postradiation survival and function of Lgr5^+^ intestinal stem cells [90]. Gene-modified MSCs are efficient in repairing acute radioactive intestinal injury [91]. At present, there are very few studies on the relationship between MSCs and gastric mucosal injury. Lrig1^+^ gastric isthmal progenitor cells have a similar ability to Lgr5^+^ intestinal stem cells [92], because it is capable of repopulating the damaged oxyntic mucosa by differentiating into normal gastric lineage cells in the mouse stomach. The activities of Lgr1^+^ gastric antrum stem cells after RIGI and their interactions with MSCs may provide some new clues for the treatment of RIGI (Fig. 3).

## FUTURE DIRECTION OF RIGI

### Summary of the diagnosis of RIGI

Radiation damage to the digestive tract has been demonstrated in many reports. Compared with the small intestine, gastric tissue lacks clinical trials and attention due to its relatively low sensitivity to radiation. However, radiation damage to the gastric mucosa also brings a lot of physical and financial burden. As the radiation beam improves and the external irradiation becomes more conformal, the irradiation volume becomes more and more reasonable. Modern RT can efficiently treat tumors while minimizing the radiotoxicity of normal tissues. The visualization of lesions on gastroscopy allows for individualized diagnosis and treatment of RIGI, but more quantifiable indicators are still needed for comprehensive risk assessment in clinical practice. Simple and readily available serum markers of gastric inflammation (gastrin and pepsinogen) are indicative of gastritis but are not limited to radiation. Dose-volume effects are key risk factors for RIGI but are not systematically applied. The classification of RIGI according to risk factors and clinical manifestations (symptoms, gastroscopy and serological inflammatory markers) and the correspondence with the Common Terminology Criteria for Adverse Events are believed to provide a more intuitive reference for the treatment of RIGI.

### Some new RT and treatment may have a benefit on RIGI

#### FLASH RT

FLASH RT is an emerging RT technology with promise, which can deliver ultrahigh rates of radiation (> 40 Gy/s) and inject the entire radiation dose into the target area to kill the tumor in a very short time (< 1 s) [93]. Compared to previous RT techniques, biological tissue undergoing FLASH RT produces a FLASH effect that is not only undiminished or even more effective in killing tumors, but also provides better protection to normal tissue. It is especially suitable for such gastric and lung that change position in real time. This effect may be due to the rapid depletion of oxygen in the target area by the ultrahigh dose transient irradiation, leaving the cells to receive the remaining dose in an anaerobic environment, so that DNA damage is limited because of the reduction of superoxide radicals generated by the reaction between free radicals and oxygen. FLASH effect has already been confirmed in several *in vivo* models. Recently, a research group extended FLASH effect to co-immunotherapy [94]. In clinical practice, a 75-year-old patient with multi-resistant CD30^+^ T-cell cutaneous lymphoma initially received FLASH RT [95]. This treatment was given to a 3.5-cm diameter skin tumor. No severe adverse events were observed during the peak of reactions and the tumor was controlled as expected. Another clinical trial used FLASH RT to treat bone metastases [96]. As a result, 50% of total patients reported a complete response and the adverse events were comparable with those of standard-of-care RT. It is worth mentioning that most FLASH RT uses electron beams with a low penetration depth (2–3 cm at most) [97]. Protons and very high-energy electrons (VHEEs) (100–200 MeV) are used to improve penetration and facilitate clinical conversion [93].

#### Mitochondrial therapy

Mitochondrial therapy involves the introduction of autologous or allogeneic mitochondria into mitochondria-damaged tissues. Exogenous mitochondria are capable of performing normal functions on substance and energy regulation, ROS production and elimination, apoptosis and anti-apoptosis, fusion-division and mitochondria-nucleus interconnection [98]. Based on these properties of exogenous mitochondria, mitochondrial therapy can be used for ischemic cardiomyopathy and other mitochondria-related diseases [99]. The pathogenesis of this disease is linked to mitochondrial structural and functional damage, as well as abnormalities in mitochondrial material and energy metabolism, which is similar to radiation-induced intracellular mitochondrial damage. Moreover, if mitochondria enter the tumor microenvironment with hypoxia and acidity, they will in turn activate the apoptosis pathway to induce tumor cell death [100, 101].

#### Exosome therapy

Exosomes are a type of cell-secreting vesicle contenting proteins, miRNAs and various kinds of lipids [102]. Unlike mitochondria, exosomes can be easily localized to the cell surface for membrane fusion or directly into the cell for lysis, as exosomes possess a large number of proteins with a high affinity for the cell membrane. Exosomal contents such as specific proteins, RNA, metabolites, etc. can be released into the cell to produce the appropriate function. MSC-derived exosomes are important extracellular communicators with regeneration-mediating and immunomodulatory effects, showing great potential in the treatment of radiation pneumonia and pulmonary fibrosis [103]. Among the methods of exosome delivering, oral administration is more effective than intravenous administration because of lower destruction by phagocytes in the liver [104]. Theoretically, oral administration does better in repairing mucosal damage and promoting intercellular communication. However, as with mitochondrial therapy, both lack the technology to be prepared in large quantities and are limited by the efficiency of the delivery system.

#### Gastric microbiota

Usually, bacteria are not easily colonized in gastric due to the extremely low pH environment. However, some sequencing results have shown a significant number of bacteria colonization in the stomach other than *H. pylori* [105, 106]. Of these, *Aspergillus* and *Bacteroides* are the dominant groups in gastric fluid samples, followed by *Phylum Thicket*, *Phylum Clostridium* and *Phylum Actinomycetes*. A 16S rRNA sequencing study using gastric mucosa as a sample [107] found dysbiosis (decrease in the absolute number and relative abundance of the dominant flora) accompanies the process from gastric inflammation to cancer development. It reveals that *Peptostreptococcus stomatis*, *Streptococcus anginosus, Parvimonas micra*, *Slackia exigua and Dialister pneumosintes* are enriched in gastric cancer. But it is still unknown whether there is a positive role for flora with reduced abundance in inflammation and cancer, and if we can screen for a beneficial bacterium that resists gastric inflammation or even has very low radiosensitivity, it should be of great help in the biological treatment of RIGI.

## CONCLUSIONS

Ultimately, the diagnosis of RIGI is based on the history of RT with consideration of target, dose, volume, as well as clinical symptoms, timing, gastroscopic findings, CT presentations. So far, there is no specific preventive measure for RIGI, and it is necessary to find suitable protective agents or methods, carefully design clinical trials and intervene before the gastric is irradiated.
